# Negative pressure pulmonary edema after percutaneous endoscopic interlaminar lumbar discectomy-a case report

**DOI:** 10.1186/s12891-018-2306-1

**Published:** 2018-11-14

**Authors:** Guo Chen, Xian-di Wang, Hong-fei Nie, Zhi-qiang Yang, Kang Chen, Zhu-hai Li, Yue-ming Song, Fu-xing Pei, Jian-cheng Zeng

**Affiliations:** 1 Department of Orthopaedic Surgery, West China Hospital, Sichuan University, N0.37 Guoxue Road, Chengdu, Sichuan Province 610041 China; 2 Department of Orthopaedic Surgery, the Affiliated Hospital of North Sichuan Medical College, No. 63 Wenhua Road, Nanchong, Sichuan Province 637000 China

**Keywords:** Percutaneous endoscopic lumbar discectomy, Negative pressure pulmonary edema, Complications

## Abstract

**Background:**

Negative pressure pulmonary edema (NPPE) is a rare complication that is more prevalent in young patients. NPPE usually results from acute upper airway obstruction, which is most commonly caused by laryngospasm during extubation. NPPE is characterized by the sudden onset of coughing, hemoptysis, tachycardia, tachypnea, and hypoxia, and is dramatically improved with supportive care, which prevents severe sequelae. To our knowledge, there is no report of a patient developing NPPE after percutaneous endoscopic interlaminar lumbar discectomy.

**Case presentation:**

Herein, we report the case of a 22-year-old amateur basketball player with L5/S1 disc herniation who developed NPPE during extubation after general anesthesia for a minimally invasive spinal surgery (percutaneous endoscopic interlaminar lumbar discectomy). The NPPE was treated by maintaining the airway patency, applying positive-pressure ventilation, administering dexamethasone and antibiotics, and limiting the volume of fluid infused. The patient had an uneventful postoperative course, and was discharged to his home on postoperative day 3.

**Conclusions:**

Although NPPE is an infrequent complication, especially in patients undergoing percutaneous endoscopic interlaminar lumbar discectomy, this case report highlights the importance of early diagnosis and prompt treatment of NPPE to prevent the development of potentially fatal complications.

## Background

Negative pressure pulmonary edema (NPPE) is an infrequent complication of acute upper airway obstruction, with an overall prevalence of 0.1% and no significant difference in the incidence between male versus female patients [1, 2]. The mechanism of NPPE is considered to involve the increased effort required to inspire against an obstructed upper airway; this exertion leads to a marked increase in negative intrathoracic pressure, an increase in normal inspiratory hemodynamic physiology, and an increase in the volume of blood that flows into the pulmonary vasculature from the systemic circulation. The increased hydrostatic pressure and lowered pericapillary interstitial pressure produce a large pressure difference between the alveoli and the capillaries, shearing the capillary membrane barrier and resulting in stress failure [1–3].

The most common causes of NPPE are near drowning, choanal stenosis, endotracheal tube obstruction, use of a laryngeal mask that causes airway obstruction or displacement, laryngeal tumor, and epistaxis. When NPPE occurs postoperatively, it is usually due to laryngospasm during extubation and postoperative vocal cord paralysis [4]. NPPE has reportedly occurred in patients undergoing many kinds of surgical procedures. In the field of orthopedics, NPPE is more likely to occur in patients undergoing spinal surgery, especially procedures involving the cervical spine [5–8]. However, there are no reports of NPPE in patients undergoing percutaneous endoscopic interlaminar lumbar discectomy (PEID). The purpose of the present case report is to highlight the possibility of NPPE during PEID, and to highlight the importance of early diagnosis and prompt treatment of NPPE to prevent the development of potentially fatal complications.

## Case presentation

A 22-year-old male amateur basketball player with no relevant medical history was admitted to our hospital due to continuous severe lower back pain with radiating nerve pain and numbness from the hip to the posterior part of the left leg. Physical examination revealed paravertebral muscle spasm, diminished sensation at the lateral aspect of the sole of the left foot, diminished strength in plantar flexion on the left side, a weakened Achilles tendon reflex on the left side, and a positive straight-leg raise test (30 degrees) on the left side. Magnetic resonance imaging of the lower spine showed a herniated nucleus pulposus at the left L5-S1 level, and so the patient was diagnosed with left lumbar disc herniation at L5-S1.

Preoperative evaluation was normal. The patient opted to undergo surgical treatment with PEID after failure of conservative treatment. Tracheal intubation was facilitated via the administration of atracurium and penehyclidine. General anesthesia was induced with propofol and fentanyl, and was maintained with sevoflurane, fentanyl, and atracurium. The PEID was successfully completed within 40 min, with complete removal of the herniated disc, annuloplasty of the annulus fibrosus, and thorough decompression of the S1 nerve root. Intraoperatively, the patient was infused with 1,100 mL of Ringer’s lactate solution. Throughout the entire procedure, the urine volume was 400 mL, and the blood loss was less than 20 mL.

Extubation was performed when the patient was conscious, spontaneously breathing, and performing purposeful movements. The patient then suddenly began to respire forcefully. The heart rate was 130 beats/min, blood pressure was 155/90 mmHg, respiratory rate was 35 breaths/min, and SpO_2_ had decreased from 98 to 65%, followed by the production of 5 ml of pink frothy sputum. Chest auscultation performed by the anesthetist revealed tachycardia and dispersed moist rales bilaterally. Arterial blood gas analysis showed that the pH was 7.34, PaO_2_ was 71 mmHg, and PaCO_2_ was 40 mmHg. Electrocardiography indicated sinus tachycardia. NPPE was diagnosed. The airway was kept unobstructed, and oxygen was delivered at 5 L/min via mask ventilation. The infusion volume was limited. Dexamethasone was administered to relieve spasm, and 20 mg of furosemide was administered intravenously to treat pulmonary edema. The SpO_2_ improved and was maintained above 93%.

**Fig. 1:**
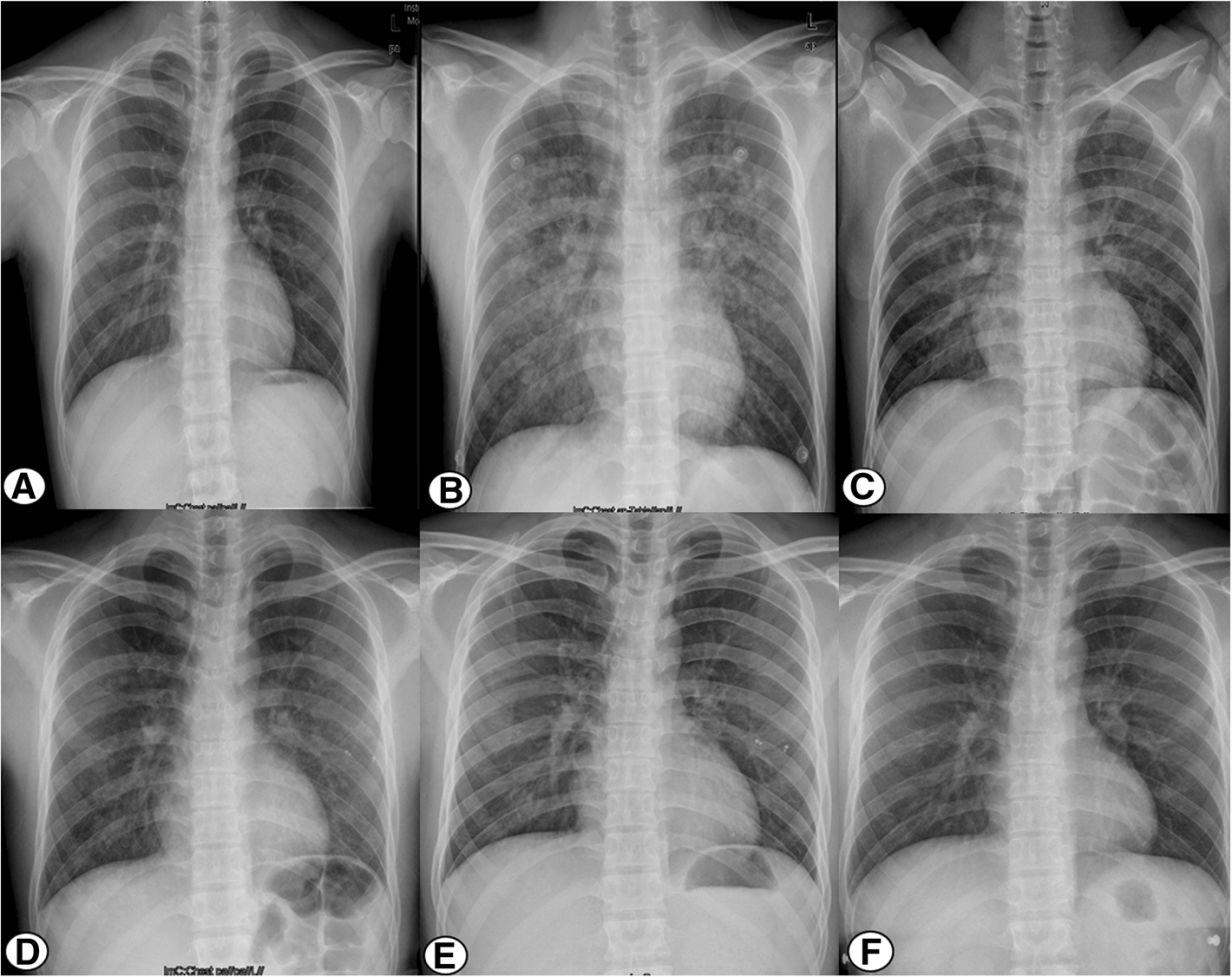
**a**-**f** chest radiograph obtain preoperative, 2 h, 17 h, 23 h, 3 days and 6 weeks after the surgery. It was shown that bilateral infiltrates with opacities and nodules gradually decreased over time and completely disappeared on follow up

**Fig. 2:**
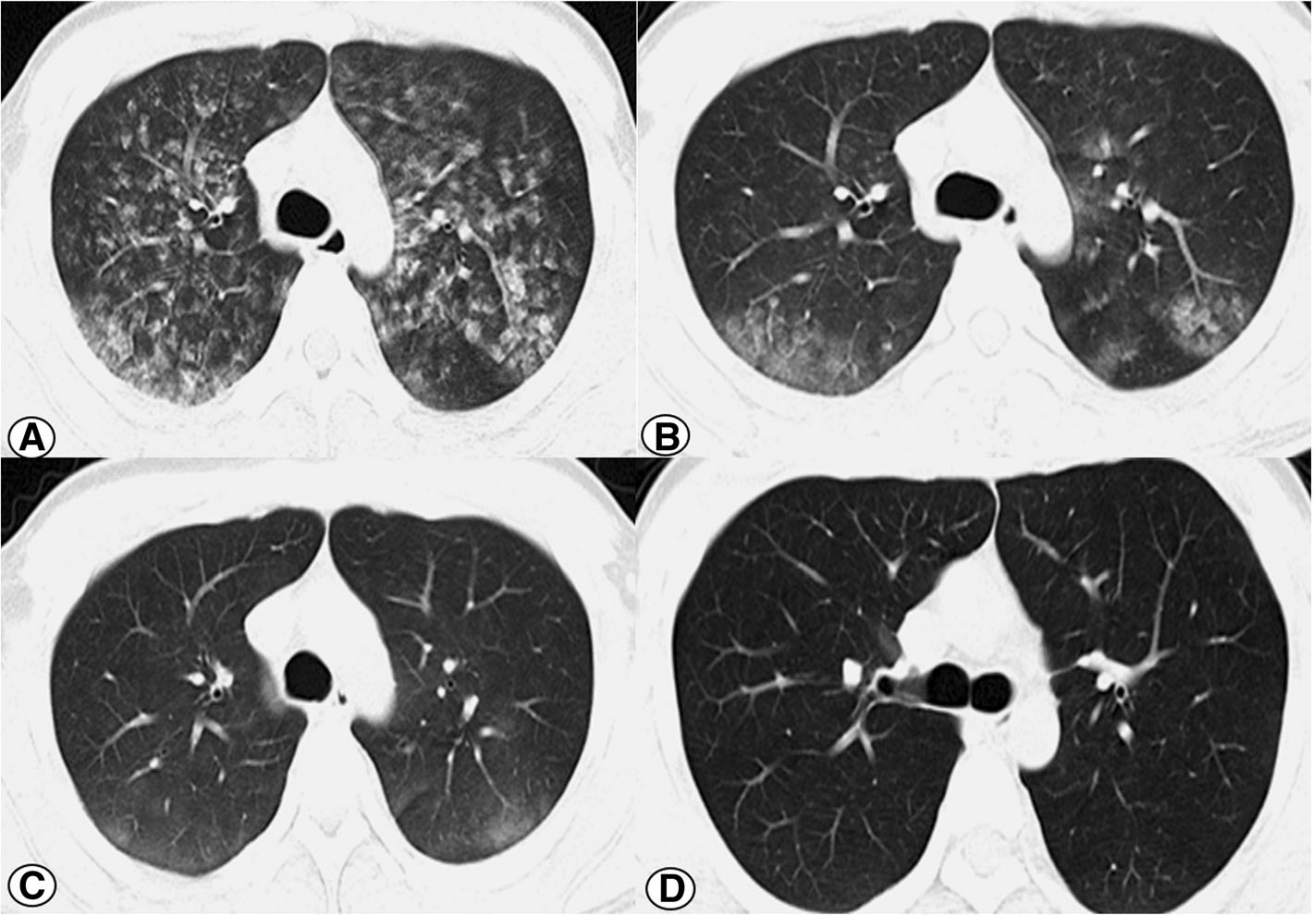
**a**-**d** Chest computed tomographic scan obtained 2 h, 25 h, 3 days and weeks after the surgery. It was shown that bilateral pleural effusion gradually decreased over time and completely disappeared on follow up

After stabilization with supportive treatment for about 60 min in the operating room, the patient was transferred to the orthopedic ward. Subsequent laboratory testing indicated an increased white cell count of 12.5 × 10^9^/L, an elevated neutrophil percentage of 84%, and a hematocrit decrease from 45.3 to 40.7%. The D-dimer level was 1.0 ng/mL, while the other main laboratory parameters were within normal limits. Repeat arterial blood gas analysis indicated that the pH was 7.37, PaO_2_ was 95 mmHg, and PaCO_2_ was 48 mmHg with oxygenation via nasal catheterization. Compared with the normal chest radiograph preoperatively (Fig. 1a), the emergency chest radiograph showed bilateral infiltrates with opacities and nodules (Fig. 1b). Two hours later, chest computed tomography (CT) of a pulmonary window showed increased vascular diameter, a mosaic pattern of attenuation, and bilateral pleural effusion (Fig. 2a).

The patient was afebrile, and cefazolin was administered prophylactically. The oxygen requirement gradually decreased to room air, with the hematocrit remaining stable, and the vital signs remaining stable for the following 24 h. A chest radiograph obtained in the morning on postoperative day 1 still showed bilateral infiltrates with opacities and nodules; however, the severity of these was obviously decreased compared with the radiograph taken the previous evening (Fig. 1c). A chest radiograph taken in the afternoon on postoperative day 1 showed that the infiltrates had further decreased. In the evening on postoperative day 1, CT showed a marked decrease in bilateral pleural effusion (Fig. 2b). The vital signs were stable, without tachycardia, tachypnea, and/or hemoptysis. At 3 days postoperatively, radiography showed nearly complete resolution of the lung infiltrate (Fig. 1d, e) and CT (Fig. 2c). The patient was discharged 3 days after spinal surgery, at which time he reported that he was satisfied with the whole therapeutic process, and had marked improvement of the symptoms in the back and left leg.

At 6 weeks postoperatively, the patient was examined in the outpatient department. The pulse oxygenation was 100% on room air, and there were no abnormal findings on chest auscultation, radiography (Fig. 1f), and CT (Fig. 2d).

## Conclusion

NPPE was first described in 1977 [1], and is an infrequent but recognized complication of upper airway obstruction. The overall prevalence of NPPE is less than 0.1% [2], with young, healthy males at greater risk compared with the general population [3]. NPPE can be caused by mechanical pressure due to hanging or strangulation [9], obstructive sleep apnea [10], aspiration of foreign material [11], endotracheal tube occlusion [12], complex intubation [13], epiglottitis and croup (especially in children) [14], and biting of the endotracheal tube [15]; one of the most common reasons for NPPE during the perioperative period is laryngospasm during extubation [2].

The mechanism of NPPE is that the exertion of inspiring against an obstructed upper airway leads to a marked increase in negative intrathoracic pressure, an increase in normal inspiratory hemodynamic physiology, and an increased volume of blood flowing into the pulmonary vasculature from the systemic circulation [1–3]. The subsequent large pressure difference between the alveoli and the capillaries shears the capillary membrane barrier and results in stress failure [2, 16, 17]. Massive sympathetic discharge triggered by hypoxia [18] and changes in intrapleural pressure [19] were also reported.

The manifestations of NPPE are stridor, suprasternal and supraclavicular retractions, desperate use of accessory respiratory muscles, panic-stricken facial countenance, decreased SpO_2_, pink frothy sputum, and crackles and wheezes on auscultation. Infiltrates in the lung with opacities and nodules may be seen on chest radiography and CT. Especially in high-risk patients, the diagnosis of NPPE can be made based on a history of a precipitating incident, typical symptoms and physical examination findings, and pulmonary edema on chest radiography. The differential diagnoses for NPPE include other causes of sudden respiratory distress, such as pneumonia, acute pulmonary edema, pulmonary embolism, and inhalation injury [17].

The treatment for NPPE should include assisted ventilation via a mask or endotracheal tube; mechanical ventilation via positive end expiratory pressure may be required if paralysis of the respiratory muscles eventuates and causes prolonged hypoxia and related complications. Patients with NPPE should have their fluid intake restricted, and dexamethasone should be administered to relieve spasm. Diuretic therapy should also be initiated to maintain a more beneficial hydrostatic balance in all capillary beds, especially in the pulmonary vasculature. In our experience, this treatment protocol results in marked improvement in most patients with NPPE, partly due to the intact of alveolar fluid clearance mechanisms.

Lumbar disc herniation is a type of spinal degeneration that generally occurs in those aged 39 to 70 years [20], with an increased incidence in those with obesity [21], a history of smoking [22], and those with specific occupations that involve sedentary behavior (such as civil servants, bank clerks, and drivers). In young patients, such as the present patient, the risk of NPPE may be increased due to genetic factors [23] and a history of trauma in daily life or during physical training [24]. Lumbar disc herniation is effectively treated by PEID, including in pediatric patients [25]. PEID is a minimally invasive procedure that has the advantages of minimal intraoperative fluoroscopy, clear exposure, complete decompression, and quick recovery with satisfactory long-term results [26–28]. We prefer to perform PEID under general anesthesia with nerve monitoring.

Young, healthy, athletic males are more susceptible to NPPE [2, 16], partly due to their relatively increased chest wall compliance and the enhanced capability to engender excessive negative intrathoracic pressure when upper airway obstruction occurs. This situation is similar to the present case in which NPPE developed in a 22-year-old amateur basketball player. Clinicians should be aware of the increased risk of NPPE in young, healthy, athletic males; caution is required when choosing anesthetic methods, and such patients should be monitored closely during extubation and the recovery period.

In our spine center, one surgical team performs more than 400 percutaneous endoscopic lumbar discectomy procedures every year (including PEID and percutaneous endoscopic transforaminal discectomy, PETD) or around six procedures every operation day. This high procedural volume involves rapid transition from one operation to the next. As PEID is minimally invasive and thus only takes a short amount of time, those undergoing PEID should be especially closely monitored to decrease the risk of NPPE; patients must be fully awake before extubation to decrease the possibility of laryngospasm, the anesthetist must provide adequate pharyngeal suctioning before extubation, and high-risk patients should be operated on early in the day to minimize the risk of anesthetist fatigue [29].

Although NPPE is an infrequent complication, especially in patients undergoing PEID, high-risk patients (young, healthy, athletic males) should be closely monitored and provided with appropriate treatment if NPPE does occur.

## Abbreviations

NPPE: Negative pressure pulmonary edema
PEID: Percutaneous endoscopic interlaminar lumbar discectomy
PETD: Percutaneous endoscopic transforaminal discectomy
